# Comprehensive Identification of *WDR* Gene Family in *Panax ginseng*: *PgWDR* Gene Expression Analysis with Ginsenosides Biosynthesis Under MeJA

**DOI:** 10.3390/biology15171516

**Published:** 2026-09-03

**Authors:** Lin Shi, Hexuan Li, Aimin Wang, Silu Zhang, Yu Zhang, Kexin Zhang, Mingzhu Zhao, Meiping Zhang, Yi Wang, Lei Zhu, Kangyu Wang

**Affiliations:** 1 College of Life Science, Jilin Agricultural University, Changchun 130118, China; nd20240994@163.com (L.S.); lihexuan@mails.jlau.edu.cn (H.L.); wangaimin@jlau.edu.cn (A.W.); zsl122221lu@163.com (S.Z.); yuzi_zhang0913@163.com (Y.Z.); zkx12302023@163.com (K.Z.); mingzhuzhao@jlau.edu.cn (M.Z.); meiping.zhang@jlau.edu.cn (M.Z.); yi.wang@jlau.edu.cn (Y.W.); 2 Jilin Engineering Research Center Ginseng Genetic Resources Development and Utilization, Jilin Agricultural University, Changchun 130118, China

**Keywords:** *Panax ginseng* C. A. Mey., WD40-repeat protein, *WDR* gene family, protopanaxadiol ginsenosides, ginsenoside biosynthesis

## Abstract

*Panax ginseng* (*Panax ginseng* C.A. Mey.) produces pharmacologically valuable ginsenosides. WD40-repeat (WDR) proteins act as versatile regulators of plant specialized metabolism, yet their biological roles under methyl jasmonate (MeJA) elicitation remain largely uncharacterized in ginseng. In this study, we identified 29 *PgWDR* family members at the whole-genome level, and systematically analyzed their phylogeny, gene structure, cis-acting promoter elements, as well as organ- and development-dependent expression patterns. Six candidate genes potentially associated with ginsenoside biosynthesis were screened through integrating gene–metabolite correlation analysis and gene co-expression analysis. Under MeJA treatment, three of these candidates showed statistically significant expression responses, while the other three exhibited variable expression fluctuations with no statistical significance. *PgWDR24* displayed a positive correlation with key ginsenoside biosynthetic enzyme genes, and a negative correlation with protopanaxadiol-type ginsenoside accumulation. Combined with its predicted nuclear localization, we hypothesize that *PgWDR24* participates in the negative modulation of protopanaxadiol-type ginsenoside accumulation, although further genetic functional validation is still required. This work provides valuable candidate genes for deciphering ginsenoside regulatory networks and offers support for molecular-assisted breeding of high-quality ginseng.

## Simple Summary

*Panax ginseng* is a valuable medicinal plant, and ginsenosides are its main active compounds. WD40-repeat proteins are known to regulate plant growth and metabolism, but their role in ginsenoside production under methyl jasmonate treatment was unclear. The *WDR* gene family was identified in ginseng and analyzed their structures, regulatory elements, and expression patterns. Six *PgWDR* genes associated with ginsenoside biosynthesis were identified via multi-dimensional screening. The expression patterns of these six genes, together with twelve key enzyme genes for ginsenoside biosynthesis that exhibited significant co-expression with the candidate genes, were further investigated in MeJA-treated ginseng adventitious roots across different treatment times. *PgWDR24* was negatively correlated with the levels of major ginsenosides (Rb1, Rb2, Rb3, Rc, and Rd), suggesting that it may be involved in negative-type association with ginsenoside accumulation.

## 1. Introduction

WD40-repeat proteins (WDR) constitute a structurally conserved and functionally diverse protein superfamily in plants, characterized by tandem arrayed WD motifs; each motif consists of approximately 40 amino acid residues and folds into four-stranded antiparallel β-sheets [1,2,3]. Such conserved structural architecture enables WDR proteins to act as versatile molecular scaffolds that mediate specific protein–protein and protein–nucleic acid interactions and facilitate the assembly of multiunit functional complexes [1]. In planta, WDR proteins participate extensively in plant growth, developmental progression, and stress adaptation by modulating signal transduction, post-translational protein modification, and abiotic stress responses [4,5,6]. In the regulatory cascade of specialized metabolism, WDR proteins precisely manipulate metabolic fluxes by forming functional complexes with transcription factors. For instance, in *Arabidopsis thaliana*, the WD40 scaffold protein TTG1 assembles a canonical MYB-bHLH-WDR (MBW) ternary complex with TT8 (bHLH) and TT2 (R2R3-MYB), which directly targets the promoter of *BAN* to activate its transcription, subsequently driving anthocyanin and proanthocyanidin biosynthesis [7]. Similarly, OsTTG1 integrates into multiple specialized metabolic regulatory circuits through interactions with diverse transcription factors, highlighting the evolutionarily conserved hub role of WD40 proteins in metabolic control [8]. Nevertheless, substantial functional divergence of WDR homologs has occurred in plant lineages. Maize SHREK1 modulates pre-rRNA processing to govern ribosome biogenesis [9]; pepper CaWD40-91 forms an unconventional MBW complex with CaAN1 and CaDYT1 to coordinately regulate anthocyanin formation and nuclear male fertility [10]; in tomato, BIW interacts with the master transcription factor BZR1 and accelerates its ubiquitination and degradation, thereby participating in brassinosteroid-governed growth and hormone signaling [11]. Despite the abundant functional documentation of the WDR family across model and crop plants, their transcriptional behaviors upon elicitation remain uncharacterized in *P. ginseng*.

*Panax ginseng* C. A. Mey. (Araliaceae) originates from Northeast Asia, including Northeast China, Korea, and the Russian Far East [12]. As one of the most precious traditional medicinal herbs in China, ginseng has been utilized clinically for thousands of years and serves as a core research material for modern pharmaceutical applications [13]. Pharmacological investigations have validated its multiple bioactivities, including anti-allergy, antioxidation, anti-tumor, and cardiovascular protection, which are mainly attributed to the abundant endogenous specialized metabolites [14,15,16,17]. Ginsenosides, the signature bioactive triterpenoid saponins of ginseng, are classified into protopanaxadiol and protopanaxatriol subtypes according to their aglycone skeletons, and the proportional content of different subtypes directly determines the commercial quality and medicinal potency of ginseng raw materials [13,18]. As intricate terpenoid metabolites, ginsenoside biosynthesis is subjected to multilayered synergistic regulation by environmental cues, endogenous phytohormone signals, and functional regulatory genes [19]. Therefore, decoding the molecular regulatory framework of ginsenoside biosynthesis and identifying pivotal regulatory genes are of great theoretical significance and practical value for ginseng cultivation optimization, new pharmaceutical development, and germplasm improvement [20].

Plants constantly confront diverse biotic and abiotic challenges in natural habitats and remodel physiological and metabolic homeostasis by triggering endogenous phytohormone signaling cascades, which synchronize stress defense, plant development, and specialized metabolic reprogramming to facilitate environmental acclimation [21]. MeJA, a pivotal lipid-derived signaling phytohormone, functions as a central integrator linking stress perception to metabolic rewiring. Exogenous MeJA application triggers intracellular hierarchical signaling cascades to reshape genome-wide transcription and drastically promotes the accumulation of high-value terpenoids and flavonoids [22]. Owing to its high induction efficiency and broad regulatory spectrum, MeJA elicitation has become a prevalent experimental strategy for dissecting metabolic regulatory mechanisms in medicinal plants [23]. Current evidence confirms a strong regulatory link between MeJA signaling and ginsenoside production. However, the response of *PgWDR* members to MeJA stimuli and their modulation of protopanaxadiol-type ginsenoside synthesis remain elusive, and a systematic regulatory network of the *PgWDR* family has not yet been established in ginseng.

In this study, we performed genome-wide identification, phylogenomic analysis, gene structure characterization, and promoter cis-element prediction combined with spatiotemporal expression profiling of the *PgWDR* family. Integrated MeJA-induced transcription quantification and gene–ginsenosides content correlation analyses were further conducted to screen candidate regulators governing protopanaxadiol ginsenoside biosynthesis and to resolve their MeJA-responsive expression signatures. Our findings provide novel genetic resources and theoretical foundations for unraveling the complex regulatory network underlying ginsenoside biosynthesis.

## 2. Materials and Methods

### 2.1. Materials and Samples

The plant materials used in this study consisted of *ginseng* plants harvested from Jilin Province at four developmental ages (5, 12, 18, and 25 years). Fourteen distinct organ types were collected, including stem, fibrous root, fruit pedicel, main root epidermis, rhizome, petiole, lateral petiolule, lateral root, branch root, leaf, fruit flesh, main root cortex, and seed. In addition, 42 root samples from locally cultivated ginseng accessions were collected and designated S1–S42. All transcriptomic datasets and ginsenoside quantification data were retrieved from our laboratory. All germplasm resources were preserved at Jilin Engineering Research Center Ginseng Genetic Resources Development and Utilization, Jilin Agricultural University.

### 2.2. Identification of the WDR Gene Family in P. ginseng

For genome-wide identification of *PgWDR* members, the hidden Markov model (HMM) corresponding to the conserved WD40 domain (Pfam accession: PF00400) was retrieved from the Pfam database (https://pfam.xfam.org, accessed on 1 March 2026). HMMER version 3.3 software was subsequently used to scan the *ginseng* proteome for preliminary candidate WDR polypeptide sequences. The retrieved candidate protein sequences were further employed as query templates to perform tBlastn version 2.1.7 searches against the in-house ginseng transcriptome database with a stringent E-value threshold of ≤1 × 10^−6^, and sequences with high homology were retained. Next, the iTAK (http://itak.feilab.net/cgi-bin/itak/index.cgi, accessed on 8 March 2026) toolkit was used to preliminarily screen redundant and false-positive candidate sequences, and non-specific candidate sequences related to transcription factors were removed. Open reading frame (ORF) prediction and full-length coding sequence (CDS) screening were accomplished using TBtools II v2.466, followed by in silico translation of nucleotide sequences into putative protein sequences matching typical WDR domain features. Conserved domain validation was independently conducted using the CD-search (https://www.ncbi.nlm.nih.gov/Structure/cdd/wrpsb.cgi, accessed on 12 March 2026) and SMART (https://smart.embl.de, accessed on 15 March 2026) online platforms. Genes carrying intact canonical WD40 domains were finally reserved and systematically designated as *PgWDR* genes.

### 2.3. Physicochemical Properties of PgWDR Proteins in Ginseng

The physicochemical properties of the deduced PgWDR proteins were calculated using the ExPASy ProtParam tool (https://web.expasy.org/protparam/, accessed on 20 March 2026). The calculated parameters included amino acid length, molecular weight (MW), theoretical isoelectric point (pI), instability index, and aliphatic index for each identified PgWDR member. The subcellular localization of all PgWDR proteins was predicted using the WoLF PSORT webserver (https://wolfpsort.hgc.jp, accessed on 24 March 2026). All the resultant data were compiled and summarized in a structured table (Table 1 and Table S1).

### 2.4. Phylogenetic, Conserved Domain, and Motif Analyses of the PgWDR Gene Family

To dissect the evolutionary relatedness and species divergence of *PgWDR* members, five plant species, including *Arabidopsis thaliana*, *Oryza sativa*, *Populus trichocarpa*, *Solanum lycopersicum*, and *Vitis vinifera*, were selected as outgroups. All retrieved WDR protein sequences were subjected to multiple sequence alignments. A maximum-likelihood (ML) phylogenetic tree was constructed using MEGA v12.0 with bootstrap replicates set at 2000, and the resulting phylogenetic tree was annotated and visualized using the iTOL webserver (https://itol.embl.de, accessed on 30 March 2026). Conserved motif prediction was performed using the MEME platform (https://meme-suite.org/meme/tools/meme, accessed on 30 March 2026), with the motif length constrained between 10 aa (minimum) and 50 aa (maximum).

### 2.5. Chromosomal Localization and Synteny Analysis of the PgWDR Gene Family in Ginseng

All identified *PgWDR* genes were mapped onto the *P. ginseng* reference genome via BLASTN version 2.1.7 searches with three filtering thresholds: sequence identity ≥ 98%, aligned length ≥ 600 bp, and E-value ≤ 1 × 10^−6^. The chromosomal distribution of *PgWDR* members was visualized using the online MG2C tool (http://mg2c.iask.in/mg2c_v2.1/index.html, accessed on 1 April 2026). Furthermore, intra-genome collinearity analysis was performed using MCScanX package in R version 4.6.0 software to identify segmental duplication-derived homologous gene pairs, and genomic synteny maps were constructed using the RIdeogram package in R version 4.6.0 software to characterize the intraspecies collinear relationships of the *PgWDR* gene family.

### 2.6. Expression Pattern Analysis of the PgWDR Gene Family in Ginseng

Transcript abundance data of candidate *PgWDR* genes were extracted from the public ginseng transcriptome database retrieved from NCBI BioProject (PRJNA302556) using R version 4.6.0 language, covering root organs from ginseng plants at four developmental ages, 14 organs of 4-year-old ginseng, and root samples derived from 42 local landraces (Table S2). Expression heatmaps were generated using TBtools II v2.466 to systematically characterize the spatiotemporal expression patterns of the *PgWDR* family.

### 2.7. Cis-Acting Element Analysis of PgWDR Gene Promoters

Based on the genome annotation and genomic localization information of *PgWDR* genes, the 2000 bp upstream regions of these genes were extracted as their promoter sequences. Cis-regulatory element prediction was performed using the PlantCARE online server (http://bioinformatics.psb.ugent.be/webtools/plantcare/html, accessed on 15 April 2026). The category, abundance, and positional distribution of the predicted motifs were statistically summarized, and the corresponding distribution heatmaps were plotted using TBtools II v2.466 to visualize the composition of the regulatory elements across individual gene promoters.

### 2.8. Co-Expression Network Analysis of the PgWDR Gene Family in Ginseng

To explore the synergistic regulatory interactions among *PgWDR* members, Spearman’s correlation coefficients were calculated using SPSS v27.0 based on transcriptomic data from 42 *ginseng* local landraces. A co-expression network was subsequently established and visualized using BioLayout Express^3D^ v3.4.

### 2.9. Correlation Analysis Between PgWDR Genes and Ginsenoside Contents

Spearman correlation analysis between PgWDR transcript abundance and the contents of the nine ginsenosides was performed using SPSS v27.0 with expression and ginsenosides content datasets from 42 ginseng landraces cultivars. Genes with significant correlations at a threshold of *p* ≤ 0.05 were screened as candidate regulators linked to ginsenoside accumulation.

### 2.10. Co-Expression Network Analysis of PgWDR Genes with Key Enzyme Genes Involved in Ginsenoside Biosynthesis

Spearman’s correlation analysis was conducted to quantify the expression correlations between *PgWDR* genes and 17 key enzyme genes involved in ginsenoside biosynthesis. The resultant correlation matrix was imported into BioLayout Express^3D^ v3.4 for co-expression network construction and visualization purposes. Integrating gene–ginsenosides and gene–enzyme correlation outcomes, candidate *PgWDR* genes associated with ginsenoside biosynthesis were hierarchically screened, laying an experimental foundation for subsequent assays on the transcriptional responses of the *PgWDR* family upon MeJA elicitation.

### 2.11. MeJA Treatment Experiment on Ginseng Adventitious Roots

Fresh healthy ginseng adventitious roots (0.5 g per sample) were inoculated in 250 mL flasks containing 125 mL of liquid B5 medium. Cultivation was performed at 22 °C in complete darkness with shaking at 110 rpm. After 20 days of pre-culture, MeJA was added to a final concentration of 200 μM for sequential harvest at 6, 12, 24, 36, 48, 60, 72, 84, and 96 h [24]. The 0h group serves as the control group. Three independent biological replicates were prepared for each treatment, and all harvested organs were immediately frozen and stored at ultra-low temperatures.

### 2.12. Expression Response Characteristics of the PgWDR Gene Family Under MeJA Treatment

Total RNA was isolated from the collected samples using Trizol reagent (CWBIO, Beijing, China), followed by cDNA synthesis using the SPARK script II reverse transcription kit (CWBIO, Beijing, China). Quantitative real-time PCR (qRT-PCR) was conducted using the 2 × Universal Blue SYBR Green qPCR Master Mix, with *PgCYP* selected as the reference gene (Table S3). The 10 μL reaction system was composed of 5.0 μL 2 × Universal Blue SYBR Green qPCR Master Mix (1 × final concentration), 0.5 μL forward primer (10 μM stock, 0.5 μM final concentration), 0.5 μL reverse primer (10 μM stock, 0.5 μM final concentration), 1 μL cDNA template, and 3 μL nuclease-free ddH_2_O. The thermal cycling program comprised an initial denaturation step at 95 °C for 30 s, followed by 40 cycles at 95 °C for 15 s and 60 °C for 30 s. Three technical replicates were arranged for each reaction, and the relative expression levels were calculated using the 2^−ΔΔCt^ method for *PgWDR* and biosynthetic enzyme genes.

One-way analysis of variance (ANOVA) implemented in SPSS version 23.0 was used for statistical significance analysis, and bar charts were generated using GraphPad Prism version 11.0 for data visualization. Spearman correlation analysis, available on the Omicsmind tools platform (https://www.omicsmind.cn/tool, accessed on 28 April 2026), was adopted to construct clustered heatmaps illustrating transcriptional correlations between *PgWDR* members and key enzyme genes under MeJA treatment for subsequent systematic evaluation.

### 2.13. Correlation Analysis Between PgWDR Genes Expression and Protopanaxadiol-Type Ginsenoside Contents Under MeJA Treatment

The ginsenoside contents of protopanaxadiol subtypes (Rb1, Rb2, Rb3, Rc, and Rd) at different MeJA treatment time points were quantified using high-performance liquid chromatography (HPLC) equipped with a Waters Alliance e2695 separation module. Spearman correlation analysis was performed on the Lianchuan OmicStudio cloud platform (https://www.omicstudio.cn/tool/59, accessed on 30 April 2026) to generate annotated clustered heatmaps depicting correlations between protopanaxadiol ginsenoside accumulation and transcript levels of key biosynthetic enzymes as well as *PgWDR* genes, followed by a comprehensive statistical analysis.

## 3. Results

### 3.1. Comprehensive Identification of the WDR Gene Family in P. ginseng

A total of 201 nucleotide sequences containing conserved WD40 domains were retrieved. Redundant and false-positive sequences were filtered out using iTAK version 1.6 software, and the remaining candidates were subjected to systematic domain validation using the ORF finder, CD-search, and SMART platforms. Based on existing studies on the protein structure of WD40 domains, a functional and intact WD40 protein needs at least four WD repeat units to assemble a complete β-propeller spatial structure, which enables it to participate in protein–protein interactions [25]. After screening, 172 of 201 sequences failed to satisfy the criteria. The remaining 29 sequences with four or more canonical WD40 motifs were retained for subsequent family characterization and were sequentially designated *PgWDR01–PgWDR29*.

### 3.2. Physicochemical Properties of PgWDR Proteins

The physicochemical properties and subcellular localization of the 29 PgWDR proteins were predicted using ExPASy ProtParam and WoLF PSORT (Table 1). The polypeptide lengths ranged from 211 to 1217 aa, with corresponding molecular weights varying from 23,271.26 to 137,124.91 Da. Theoretical pI values ranged from 4.85 to 9.23: approximately 69% of the proteins had a pI below 7 and were acidic, and the remaining were basic proteins. The instability index fluctuated between 20.12 and 53.65, respectively. Twenty-two proteins with an instability index < 40 were classified as stable, and the other seven with values ≥ 40 were classified as unstable. All PgWDR proteins had an aliphatic index higher than 50 (53.55–94.43), indicating generally robust thermal stability. Subcellular localization prediction assigned these proteins to five compartments: 13 in the nucleus, nine in the Cytoplasm, five in chloroplasts, one in peroxisomes, and one in the endoplasmic reticulum.

### 3.3. Phylogenetic Tree Analysis of the PgWDR Gene Family

**Figure 1:**
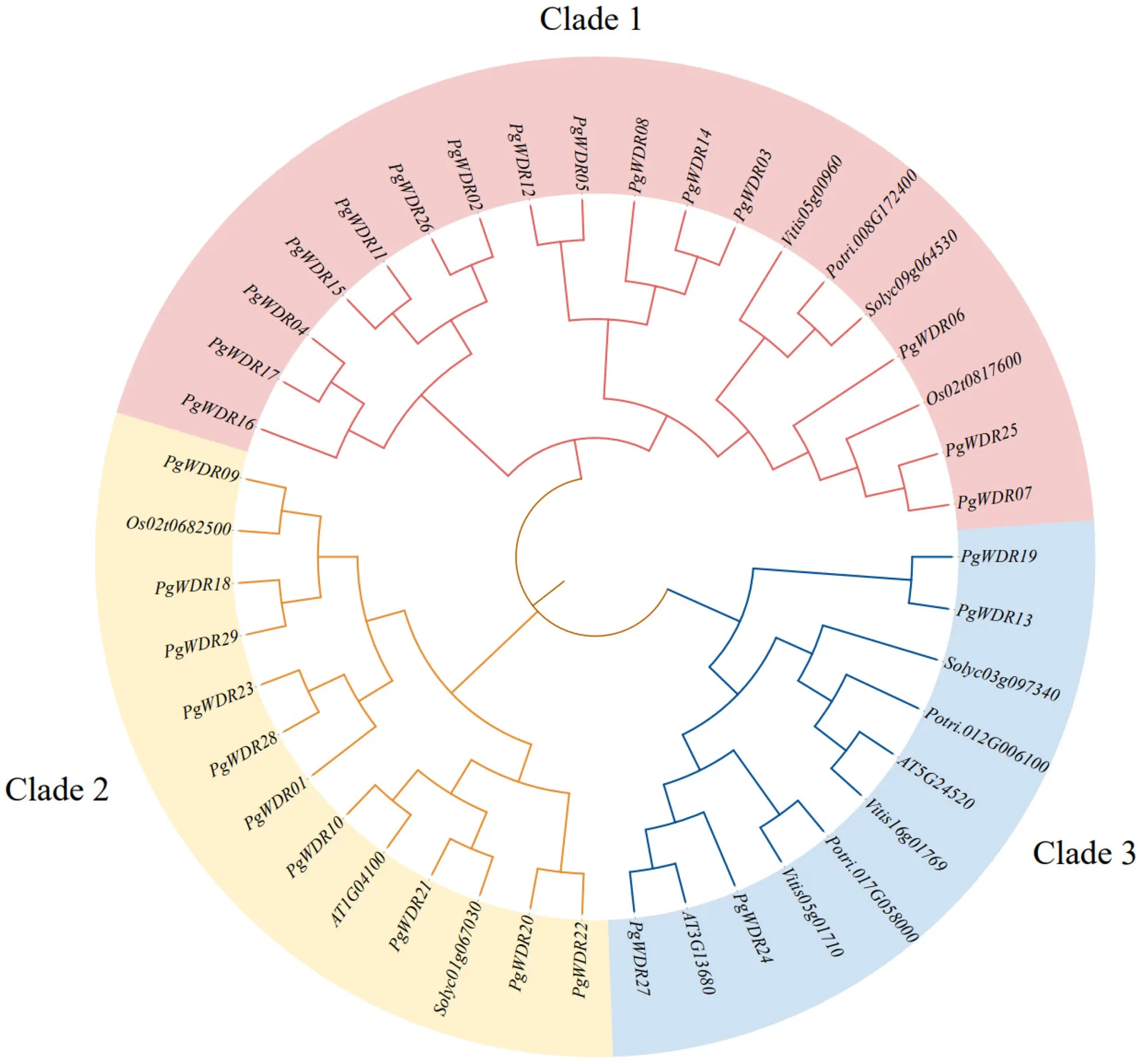
Phylogenetic tree of the *PgWDR* gene family and WDR homologs from other plant species. Orthologous WDR genes from *A. thaliana* (AT), *O. sativa* (Os), *S. lycopersicum* (Solyc), *V. vinifera* (Vitis) and *P. trichocarpa* (Potri) were used as out-group sequences. The *PgWDR* family is divided into three main clades, each labeled with a distinct background color, pink is Clade 1, yellow is Clade 2, and blue is Clade 3.

To clarify the evolutionary relatedness of the *PgWDR* family, a maximum-likelihood (ML) phylogenetic tree was constructed using the 29 identified *PgWDR* sequences together with homologous WD40 proteins from five outgroup species (*A. thaliana*, *O. sativa*, *P. trichocarpa*, *S. lycopersicum*, and *V. vinifera*). As presented in Figure 1, the *PgWDR* family was divided into three distinct clades (Clades 1, 2, and 3). Clade 1 was the largest subgroup, consisting of 15 *PgWDR* members, which clustered closely with homologs from grape, tomato, poplar, and rice and exhibited high evolutionary conservation. Clade 2 contained 10 *PgWDR* genes and formed a ginseng-specific monophyletic branch. The four members in Clade 3 shared a closer phylogenetic affinity with their counterparts from *Arabidopsis*, poplar, and grape, implying strong conservation across dicotyledonous species.

### 3.4. Conserved Motif and Domain Analysis of the PgWDR Gene Family

**Figure 2:**
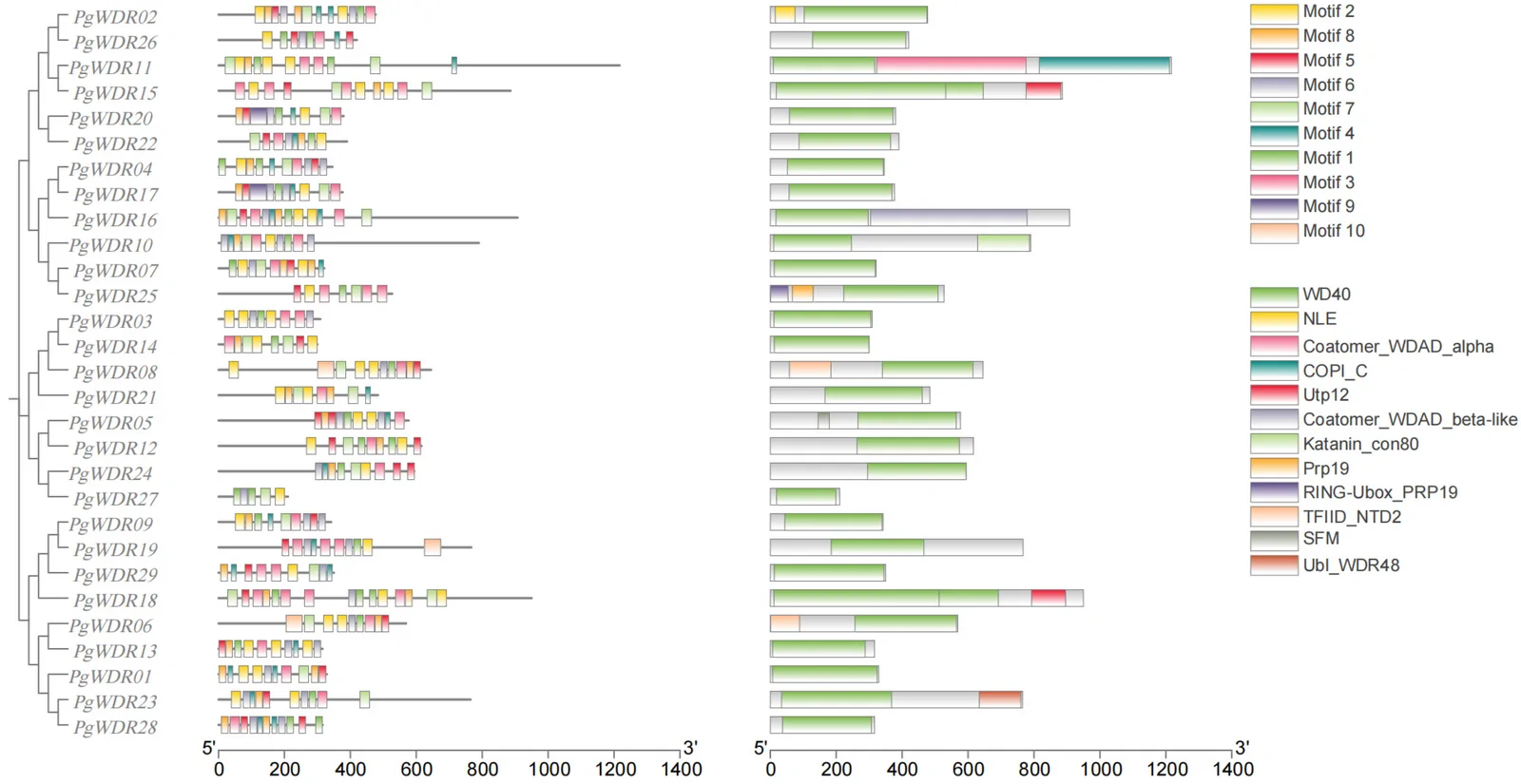
Phylogenetic relationships, conserved motifs and domain architectures of PgWDR proteins. Fonts in distinct colors correspond to diverse subfamilies, while colored boxes denote conserved motifs and functional domains.

Conserved motif and functional domain analyses were performed on the 29 PgWDR proteins to further characterize their classification and functional divergence of the *PgWDR* family. As illustrated in Figure 2, the middle panel exhibits the distribution of ten conserved motifs (Motif 1–10) predicted by MEME, with distinct colors indicating individual motifs. The right panel displays the annotated functional domains colored differentially according to the domain type. All PgWDR proteins harbored canonical core WD40 domains, and members within the same phylogenetic clade shared highly consistent motif compositions, corroborating the reliability of phylogenetic grouping. Variable accessory domains were detected among proteins from different clades, indicating functional diversification during evolutionary progression.

### 3.5. Chromosomal Localization and Synteny Analysis of the PgWDR Gene Family

**Figure 3:**
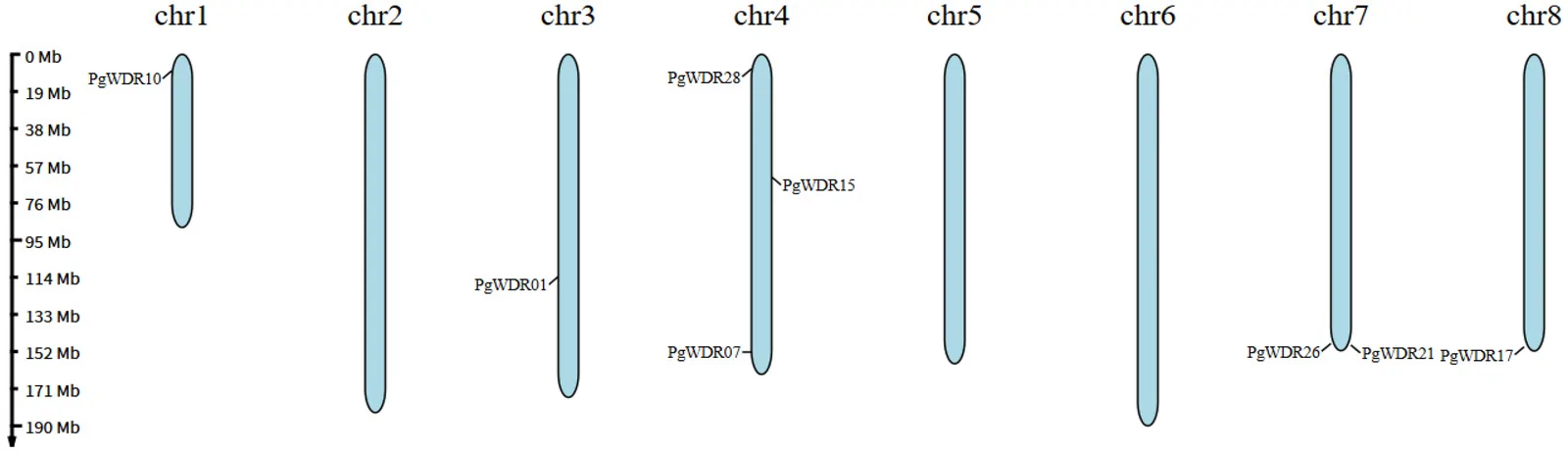

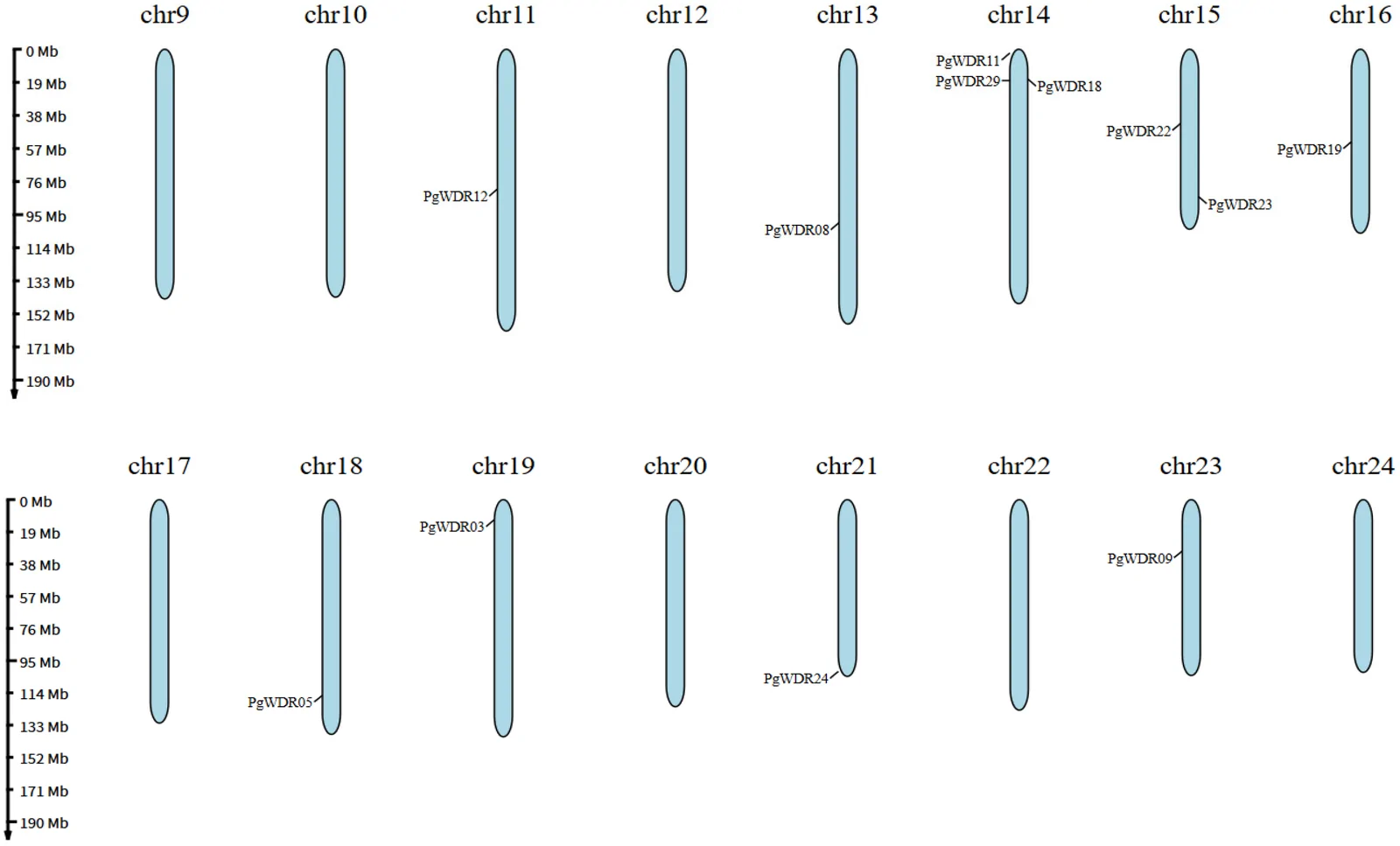
Chromosomal distribution of *PgWDR* genes in *P. ginseng*. Chromosome numbers (chr1–chr24) are marked atop each chromosome, and the left-side scale bar represents chromosome length (Mb). Genes are mapped to their physical locations on chromosomes.

**Figure 4:**
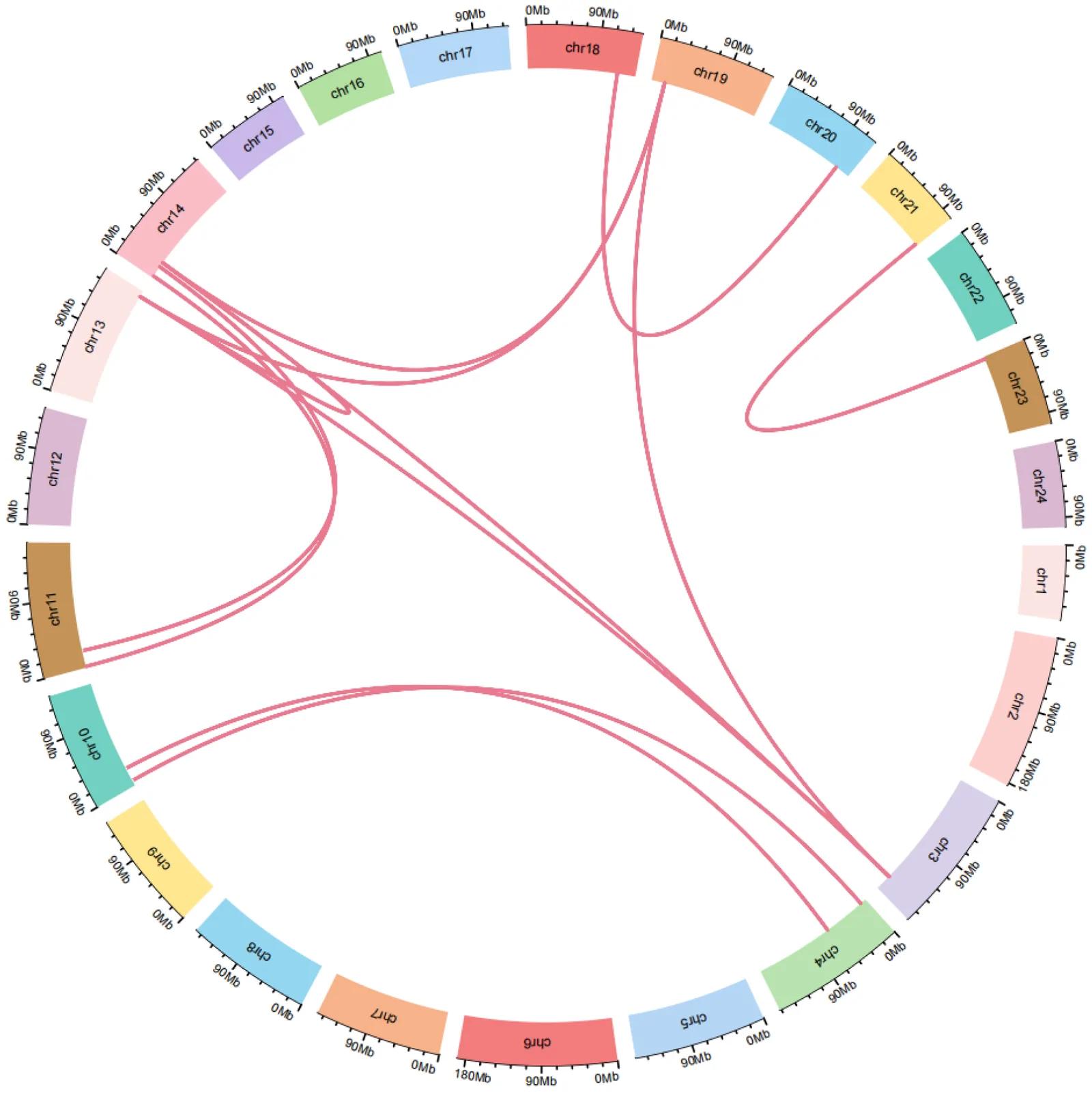
Intragenomic collinearity of *PgWDR* genes in *P. ginseng*. Pink arcs connect collinear gene pairs derived from gene duplication; colored blocks stand for ginseng chromosomes, and peripheral scales denote chromosomal physical length. Mb, megabase.

Chromosomal localization and synteny analyses of the 29 *PgWDR* genes were conducted using the ginseng reference genome to uncover their genomic arrangement and family expansion mechanisms. Chromosomal mapping revealed that 20 out of 29 *PgWDR* genes were unevenly scattered across 14 of the 24 ginseng chromosomes, whereas nine *PgWDR* genes (*PgWDR02*, *PgWDR04*, *PgWDR06*, *PgWDR13*, *PgWDR14*, *PgWDR16*, *PgWDR20*, *PgWDR25*, and *PgWDR27*) could not be anchored to any chromosome, because they reside on unanchored scaffolds of the present ginseng reference genome. This phenomenon is frequently observed for species with large, highly heterozygous genomes such as *Panax ginseng*, where some contigs cannot be reliably placed onto chromosomes during genome assembly (Figure 3). Chromosomes chr4 and chr14 each harbored three *PgWDR* genes and possessed the highest gene density (Figure 4); chr1, chr3, chr7, chr8, chr11, chr13, chr15, chr16, chr18, chr19, chr21, and chr23 contained one or two copies apiece, and no *PgWDR* homologs were identified on the remaining chromosomes. Several genes clustered closely on particular chromosomes, such as *PgWDR07/15/28* on chr4 and *PgWDR11/18/29* on chr14, suggesting probable tandem duplication events contributed to *PgWDR* family expansion. Both tandem and segmental duplication events have participated in the expansion of the *PgWDR* gene family.

### 3.6. Expression Pattern Analysis of the PgWDR Gene Family

**Figure 5:**
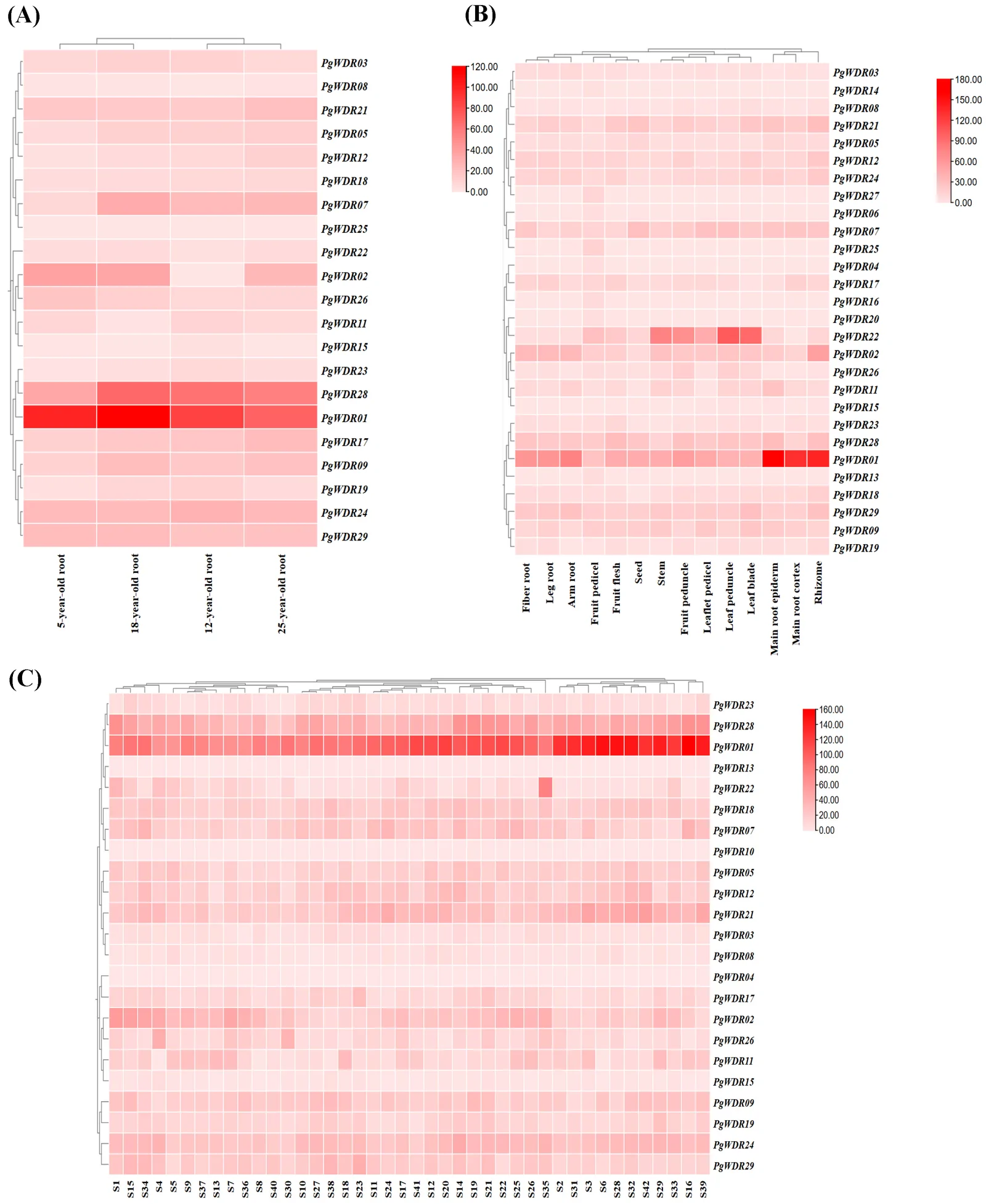
Heatmap spatiotemporal expression profiles of *PgWDR* genes in *P. ginseng*. (**A**) Gene expression of *PgWDR* genes in 4 different ages (5, 12, 18, 25 years old) of ginseng roots. (**B**) Gene expression of *PgWDR* genes in 14 different organs of 4-year-old ginseng. (**C**) Gene expression of *PgWDR* genes in 42 farmer cultivars of 4-year-old ginseng roots.

In this study, the spatiotemporal expression profiles of the *PgWDR* family were systematically characterized. For ginseng roots of four cultivation ages (Figure 5A), *PgWDR01* and *PgWDR28* maintained constitutively high expression across 5- to 25-year-old plants, peaking at the 18-year stage. Several other genes, including *PgWDR07* and *PgWDR18*, exhibited stage-specific upregulation, implying their potential regulatory roles in specialized metabolism during the late ginseng developmental stages. Organ-specific transcript patterns (Figure 5B) revealed a pronounced preferential expression among *PgWDR* members. Specifically, *PgWDR01* was predominantly expressed in underground organs, whereas *PgWDR22* accumulated exclusively in floral organs, supporting the functional divergence of paralogs in distinct biological pathways in plants. Expression profiling across 42 local ginseng landraces (Figure 5C) demonstrated that *PgWDR01* and *PgWDR28* were consistently highly transcribed, with negligible transcriptional variation among all the accessions. In contrast, a subset of *PgWDR* genes displayed cultivar-biased expression; for instance, *PgWDR22* was drastically upregulated only in accession S35 but maintained low transcript abundance in the remaining genotypes, highlighting divergent transcriptional regulation shaped by varied genetic backgrounds.

### 3.7. Promoter Cis-Acting Element Analysis of the PgWDR Gene Family

**Figure 6:**
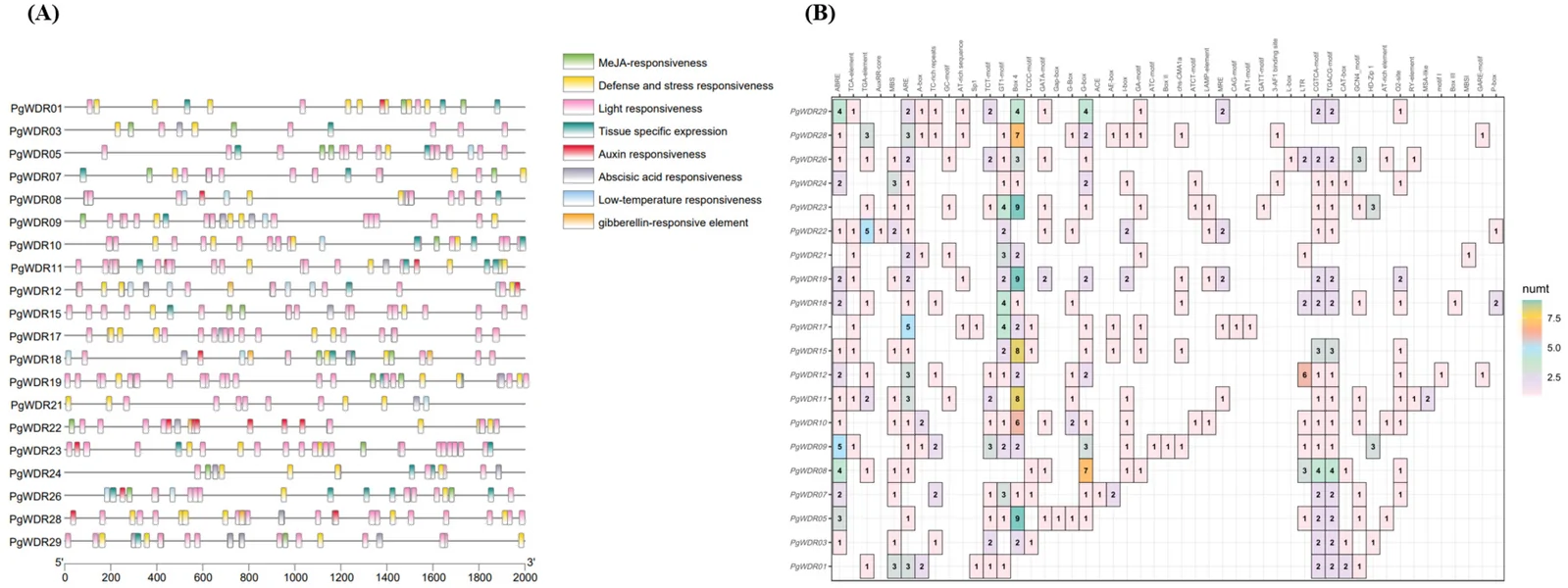
Cis-acting element analysis of the *PgWDR* genes promoters in *P. ginseng*. (**A**) Analysis of cis-acting elements of the *PgWDR* gene family. Colored circles represent cis-acting elements with different functions. (**B**) Heatmap for cis-acting regulatory elements in *PgWDR* genes.

Considering that nine *PgWDR* genes are located on unanchored scaffolds with no available flanking genomic sequences, only the remaining 20 chromosomally anchored *PgWDR* genes were included in our cis-element analysis. As summarized in Figure 6A, abundant cis-elements were widely distributed across nearly all *PgWDR* promoters, which were functionally grouped into three principal categories: (1) hormone-responsive motifs, including the abscisic acid-responsive ABRE, methyl jasmonate-responsive CGTCA-motif, and auxin-responsive AuxRE; (2) stress-related regulatory elements such as low-temperature-responsive LTR, defense- and stress-responsive TC-rich repeats, and light-responsive G-box; and (3) development-associated elements covering organ-specific and meristem-specific expression motifs. In Figure 6B, cells with divergent colors correspond to varying element abundances, with embedded numerals indicating the exact counts of individual cis-motifs. Discrepancies in the category, quantity, and genomic distribution of cis-elements were observed among distinct *PgWDR* promoters. These results suggest that *PgWDR* family members participate in ginseng growth, development, and stress adaptation by perceiving diverse endogenous phytohormone cues and exogenous environmental factors.

### 3.8. Co-Expression Network Analysis of the PgWDR Gene Family

**Figure 7:**
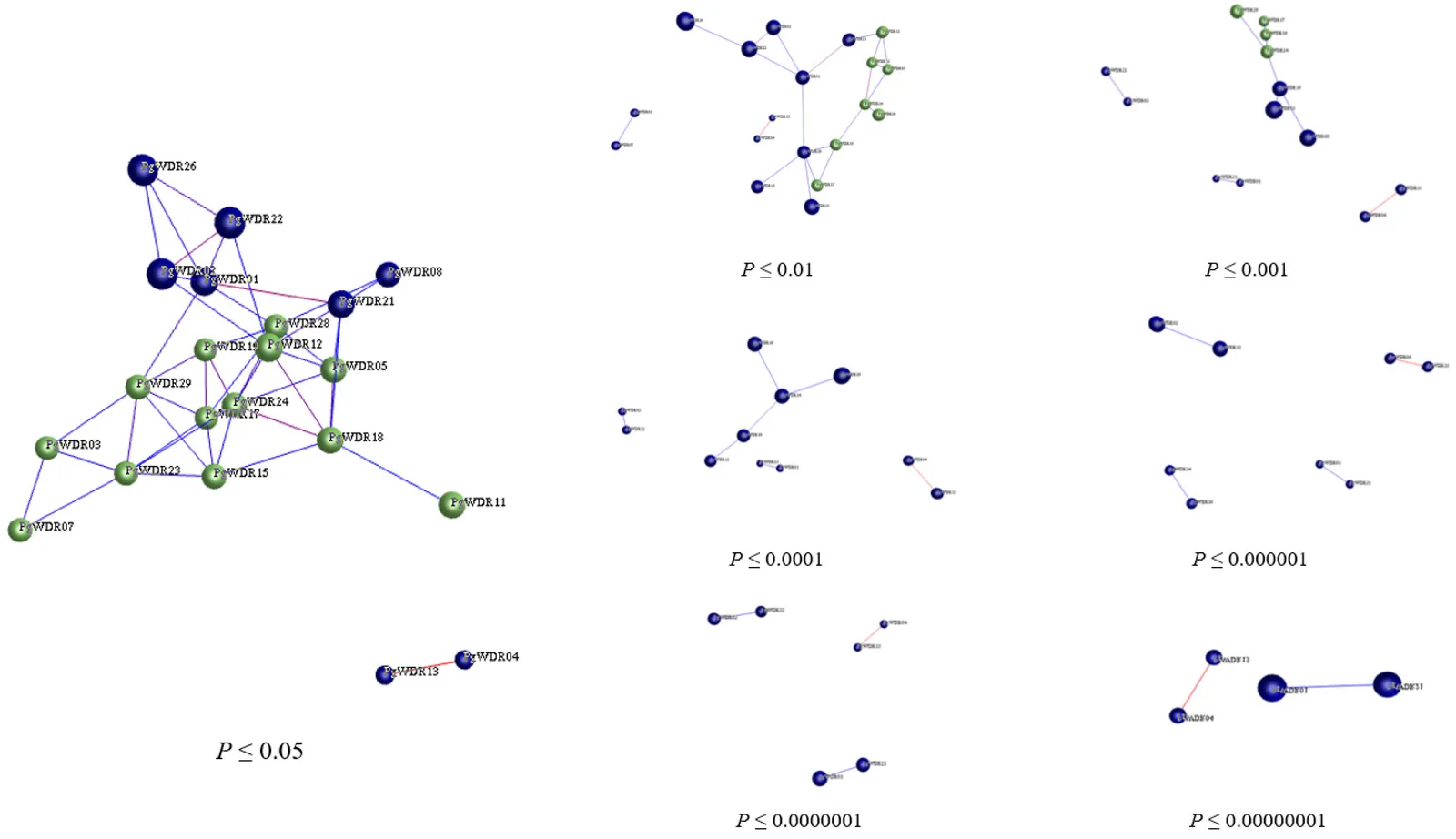
Co-expression network of *PgWDR* genes expressed in 42 local cultivars.

A co-expression network was constructed based on transcriptome datasets to dissect the potential regulatory interplay among *PgWDR* family members (Figure 7). Network analysis revealed that several *PgWDR* genes clustered into tightly connected co-expression modules, among which *PgWDR01*, *PgWDR18*, and *PgWDR28* acted as hub nodes and exhibited significant correlations with numerous other family members (*p* ranging from ≤0.05 to ≤1 × 10^−7^). Green nodes denote hub genes with high connectivity, and blue nodes represent ordinary genes. Links with varying significance levels reflect the magnitude of co-expression relationships. Collectively, these results indicate that *PgWDR* members function synergistically rather than independently to modulate biological processes, offering valuable clues for subsequent functional validation of pivotal regulatory candidates.

### 3.9. Screening of PgWDR Candidate Genes Significantly Associated with Ginsenoside Biosynthesis

The Spearman correlation between the transcript abundances of *PgWDR* genes and the contents of nine ginsenosides (Rg1, Re, Rf, Rb1, Rg2, Rc, Rb2, Rb3, and Rd) was calculated, employing expression and ginsenosides content data from 42 ginseng landraces cultivars. Eleven *PgWDR* genes (*PgWDR02*, *PgWDR03*, *PgWDR07*, *PgWDR08*, *PgWDR15*, *PgWDR19*, *PgWDR21*, *PgWDR22*, *PgWDR23*, *PgWDR24*, and *PgWDR29*) displayed statistically significant correlations with ginsenoside accumulation (*p* ≤ 0.05). Subsequently, a co-expression network linking *PgWDR* candidates and known key enzyme genes involved in ginsenoside biosynthesis was established. Ten *PgWDR* members (*PgWDR01*, *PgWDR02*, *PgWDR12*, *PgWDR18*, *PgWDR19*, *PgWDR21*, *PgWDR22*, *PgWDR24*, *PgWDR28*, and *PgWDR29*) were significantly co-expressed with 12 encoding key enzymes genes in the ginsenoside biosynthetic pathway, including *CYP716A52v2_3*, *CYP716A53v2_1*, *DS_1*, *DS_3*, *FPS_22*, *SE2_4*, *CYP716A47_1*, *SE2_1*, *SS_1*, *UGTPg71A29*, *PgURT94*, and *UGT74AE2*. By integrating the association results of ginsenoside-related genes and enzyme-related genes, we identified six core candidate regulators (*PgWDR02*, *PgWDR19*, *PgWDR21*, *PgWDR22*, *PgWDR24*, and *PgWDR29*) that are involved in ginsenoside biosynthesis. Complete gene information from the two screening sets is summarized in Table S4.

### 3.10. PgWDR Candidate Genes Expression Analysis Under MeJA Induction

**Figure 8:**
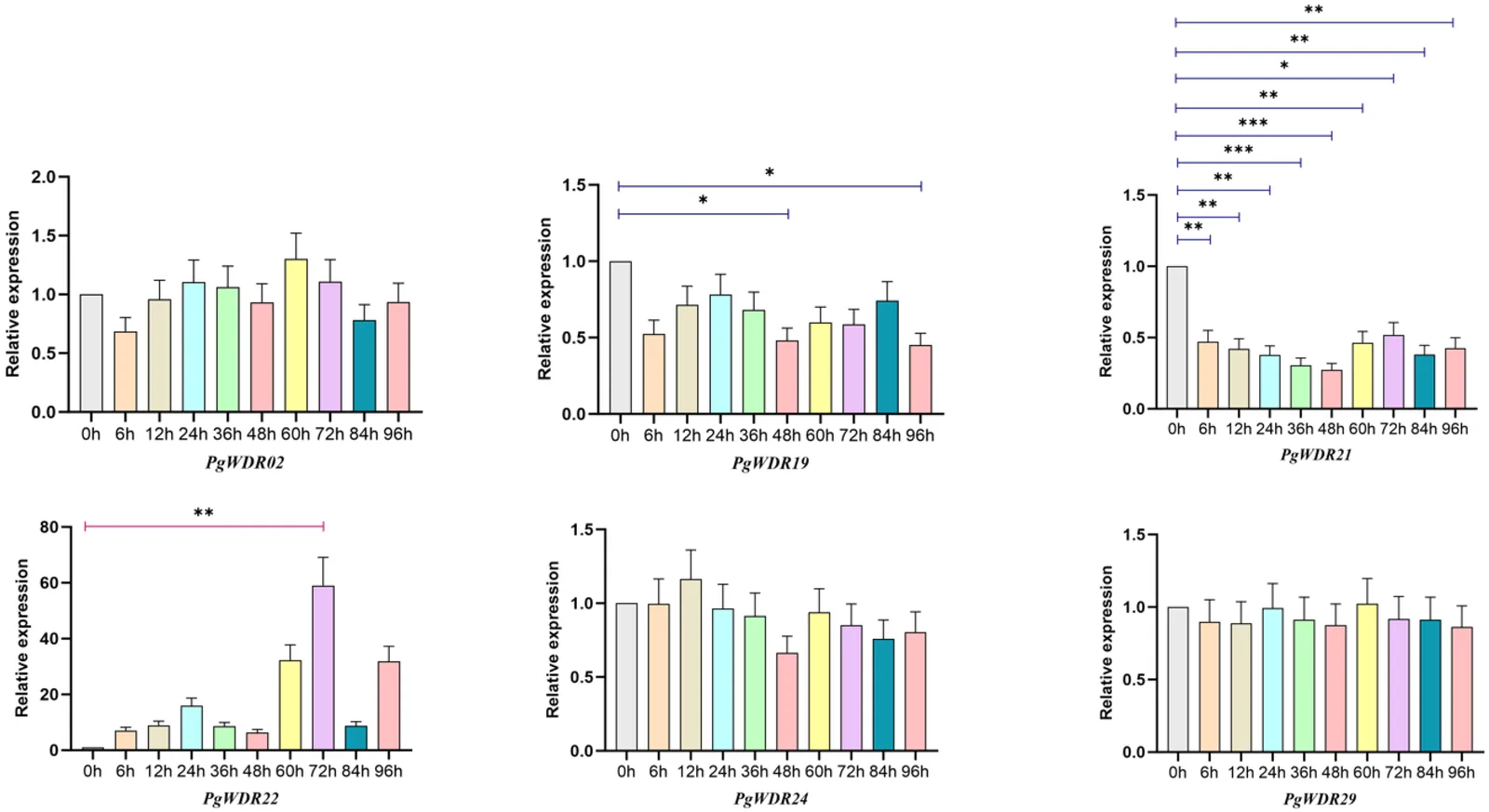
The expression of six *PgWDR* genes was detected under MeJA treatment of ginseng adventitious roots. The gene expression of *PgWDR02*, *PgWDR19*, *PgWDR21*, *PgWDR22*, *PgWDR24*, and *PgWDR29* genes were analyzed by qRT-PCR at different treatment times. The gene expression at 0 h was set as “1” to calculate the relative expression of the six genes, values were the average of three replicates. “*” indicated significant difference at *p* ≤ 0.05, “**” indicated significant difference at *p* ≤ 0.01, “***” indicated significant difference at *p* ≤ 0.001, respectively.

To study the expression responses of the *PgWDR* family to MeJA elicitation, the dynamic expression patterns of candidate *PgWDR* genes and ginsenoside-biosynthetic key enzyme genes were quantified across MeJA treatment time-series (Figure 8). These data show that MeJA treatment inhibits the expression of *PgWDR19* and *PgWDR21*, while significantly upregulating the expression of *PgWDR22*, and this regulatory effect is especially prominent after 72 h of treatment.

**Figure 9:**
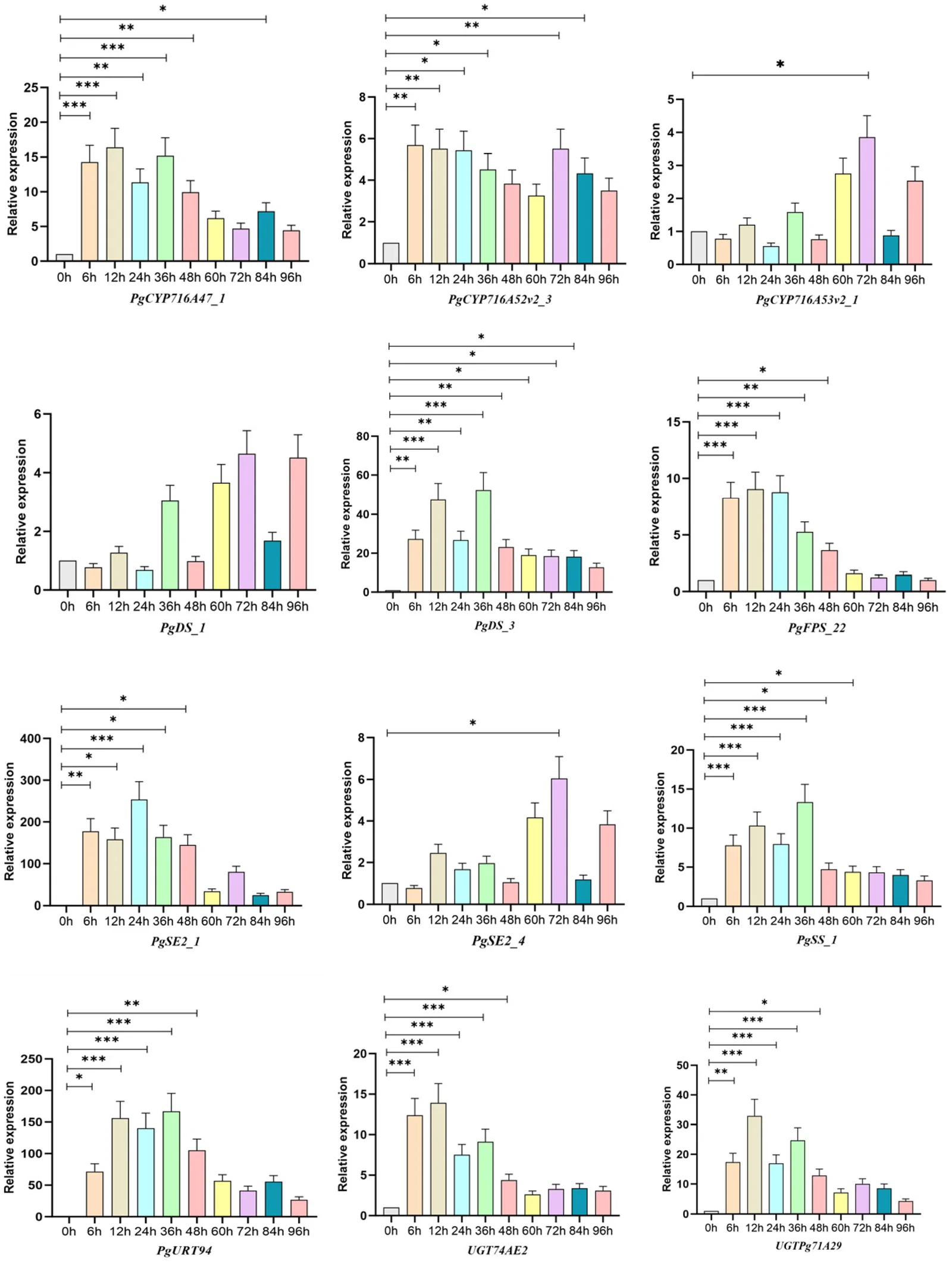
The expression of twelve key enzyme genes in ginsenoside biosynthesis was detected under MeJA treatment of ginseng adventitious roots. The gene expressions of twelve key enzyme genes were analyzed by qRT-PCR at different treatment times. The gene expression at 0 h was set as “1” to calculate the relative expression of the twelve key enzyme genes, values were the average of three replicates. “*” indicated significant difference at *p* ≤ 0.05, “**” indicated significant difference at *p* ≤ 0.01, “***” indicated significant difference at *p* ≤ 0.001, respectively.

Among the 17 initially collected enzyme genes, 12 showed significant co-expression with the six candidate *PgWDR* genes, and these 12 genes were also significantly correlated with ginsenoside content. Therefore, the 12 enzyme genes were selected as research subjects for subsequent analysis of expression profiles induced by MeJA. As illustrated in Figure 9, 11 of the 12 ginsenoside-synthetic enzyme genes were drastically upregulated under MeJA treatment, except *PgDS_1*, with distinct temporal expression patterns, confirming that exogenous MeJA effectively triggers the transcription of core enzyme genes responsible for ginsenoside biosynthesis.

### 3.11. Correlation Analysis Between PgWDR Genes and Key Enzyme Genes Involved in Ginsenoside Biosynthesis

**Figure 10:**
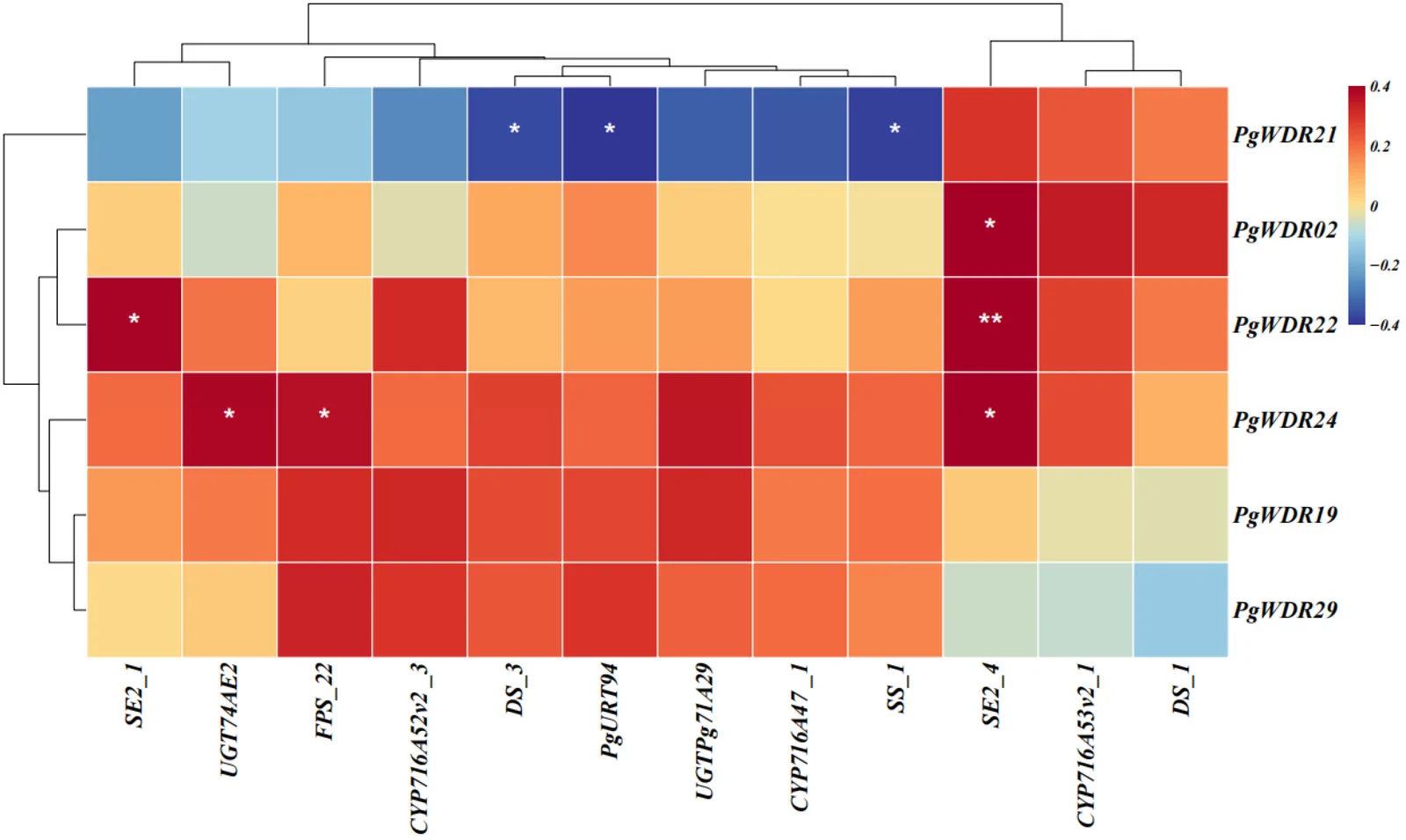
Correlation analysis of expression levels between six *PgWDR* candidate genes and twelve key enzyme genes involved in ginsenoside synthesis. The clustered heatmap of expression correlation was constructed using the Spearman correlation algorithm on the LC-BIO Cloud Platform. “*” indicates significant correlation at *p* ≤ 0.05, “**” indicated significant difference at *p* ≤ 0.01.

Spearman’s rank correlation analysis based on time-series expression data under MeJA elicitation was performed to construct a correlation heatmap between the six candidate *PgWDR* genes and 12 ginsenoside biosynthetic enzyme genes, aiming to uncover their co-expression regulatory interactions and screen master regulatory candidates (Figure 10). Functional divergence and varied correlation coefficients were observed between these genes, with multiple statistically significant gene pairs identified.

*PgWDR24* was significantly positively correlated with *FPS_22*, *UGT74AE2*, and *SE2_4* (*p* ≤ 0.05). *PgWDR22* exhibited a significant positive correlation with *SE2_1* (*p* ≤ 0.05) and an extremely significant positive correlation with *SE2_4* (*p* ≤ 0.01). *PgWDR02* was markedly positively associated with *SE2_4* (*p* ≤ 0.05). In contrast, *PgWDR21* was significantly negatively correlated with *DS_3*, *PgURT94*, and *SS_1* (*p* ≤ 0.05). No statistically meaningful correlations were detected between *PgWDR19* and *PgWDR29*, any of the tested enzyme genes; only weak non-significant correlative tendencies existed.

### 3.12. Correlation Analysis Between PgWDR Gene Expression and Protopanaxadiol-Type Ginsenoside Contents Under MeJA Treatment

**Figure 11:**
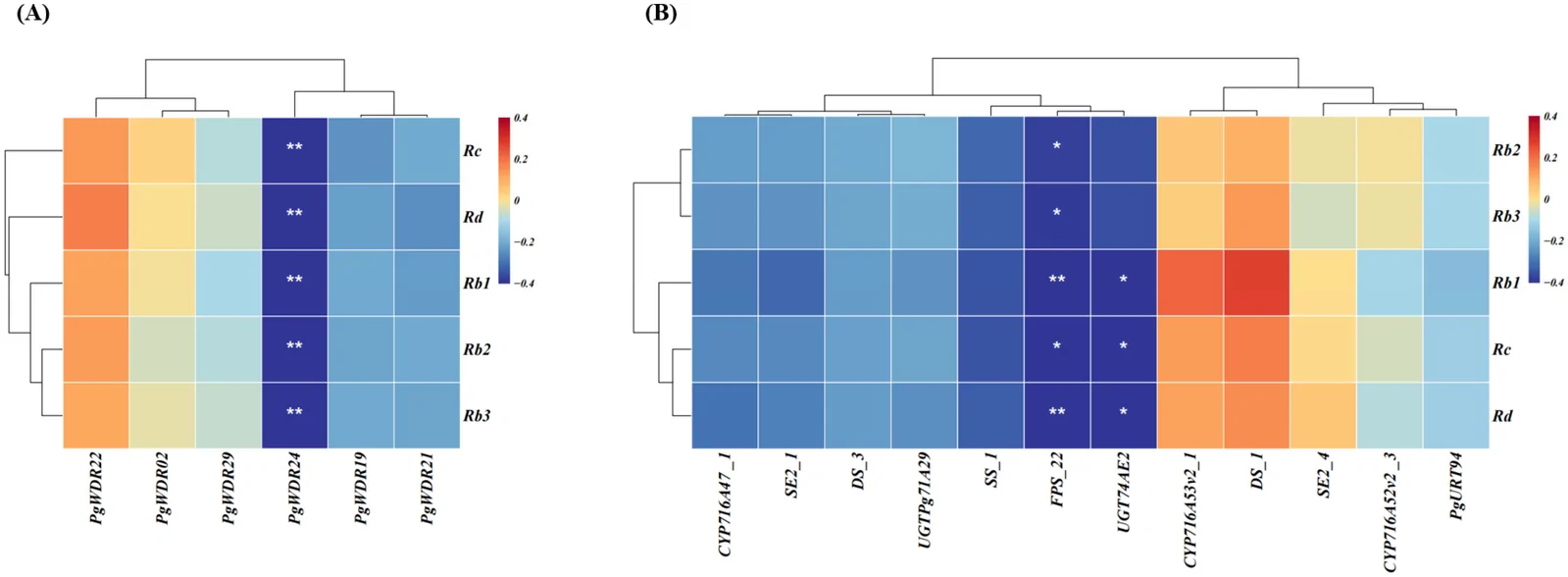
Correlation analysis of six *PgWDR* candidate genes and twelve key enzyme genes involved in ginsenoside biosynthesis expression levels with five ginsenoside contents (Rc, Rd, Rb1, Rb2, and Rb3) under MeJA induction. (**A**) Correlation analysis of six *PgWDR* candidate genes expression levels with five ginsenoside contents. (**B**) Correlation analysis of twelve key enzyme genes involved in ginsenoside biosynthesis expression levels with five ginsenoside contents. The clustered correlation heatmap was constructed using the Spearman correlation algorithm via the LC-BIO Cloud Platform. “*” indicates significant correlation at *p* ≤ 0.05, “**” indicated significant difference at *p* ≤ 0.01, respectively.

To verify the regulatory roles of *PgWDR* candidate genes in protopanaxadiol-type ginsenoside biosynthesis, Spearman’s rank correlation analysis was performed using transcript abundance and ginsenosides content quantification data obtained after MeJA elicitation. Annotated clustered heatmaps were generated to decipher the correlations between candidate *PgWDR* genes and the five protopanaxadiol ginsenosides (Figure 11A). Statistical analysis revealed that only *PgWDR24* exhibited a highly significant negative correlation with all detected protopanaxadiol ginsenosides, whereas the other five *PgWDR* genes showed no meaningful correlations with ginsenoside content (*p* > 0.05).

As summarized in Figure 11B, *FPS_22* displayed a highly significant negative association with Rb1 and Rd (*p* ≤ 0.01) and significant negative correlations with Rb2, Rb3, and Rc (*p* ≤ 0.05). *UGT74AE2* was significantly negatively correlated with Rb1, Rc, and Rd (*p* ≤ 0.05). No other tested enzyme genes had statistically significant relationships with protopanaxadiol ginsenoside content. Collectively, these observations indicate that *PgWDR24* may be associated with reduced protopanaxadiol-type ginsenoside accumulation, but its potential regulatory relationship remains to be confirmed experimentally.

## 4. Discussion

In the present study, we performed the first genome and transcriptome identification of the *WDR* gene family in *P. ginseng* and systematically characterized their expression patterns and potential links associated with protopanaxadiol-type ginsenoside biosynthesis in response to MeJA elicitation. Our transcript-level correlation analysis indicates that *PgWDR24* shows a mild expression trend under MeJA treatment, although this expression change did not reach statistical significance. The transcript level of *PgWDR24* is positively correlated with that of multiple key ginsenoside biosynthetic enzyme genes, but it is significantly negatively correlated with the accumulation of protopanaxadiol-type ginsenosides. Nevertheless, its potential regulatory relationship remains to be experimentally determined. These findings provide a novel regulatory candidate and theoretical foundation for elucidating the molecular regulatory machinery of ginsenoside biosynthesis.

A total of 29 *PgWDR* genes were identified and clustered into three distinct clades using phylogenetic analysis. Members within the same clade share highly conserved motif compositions, whereas divergent auxiliary domains exist among different clades, implying functional divergence of *PgWDR* genes in secondary metabolism, stress adaptation, and fundamental physiological processes. Such evolutionary features are consistent with previously characterized *WDR* families in tomato [26], rice [27], sugar beet [28], woodland strawberry [29], and walnut [30]. Chromosomal localization and synteny analyses revealed that both tandem and segmental duplication events contributed to the expansion of the *PgWDR* gene family in *Panax ginseng*. Closely arranged gene clusters on several chromosomes indicated the contribution of tandem duplication, while collinearity analysis revealed that segmental duplication also played an important role in this gene family. For instance, previous studies on *WDR* genes in *Rhododendron simsii* [31], mango [32], and sugarcane [33] have validated the dominant contribution of segmental duplication to family expansion and functional differentiation. Promoter cis-element prediction demonstrated that most *PgWDR* promoters harbor abundant MeJA-responsive, stress-related, and hormone-regulatory motifs, which provide a molecular basis for their participation in MeJA-triggered ginsenoside metabolic modulation [22,23].

The *PgWDR* family exhibits pronounced organ-preferential and age-dependent expression. *PgWDR01* and *PgWDR28* were persistently highly expressed in aged ginseng roots, implying their potential functions in root development and homeostasis in secondary metabolism. The high transcript abundance of *PgWDR22* in floral organs suggests its involvement in reproductive development. Plant WD40 proteins are known to exert their functions by forming various protein complexes. Co-expression network analysis identified *PgWDR01*, *PgWDR18*, and *PgWDR28* as hub genes that form tight synergistic regulatory modules with other family members, demonstrating that *PgWDR* genes cooperatively execute biological functions rather than independently [7,34].

Accumulating evidence has proven that MeJA modulates secondary metabolism via the canonical JAZ-MYC2 cascade and markedly upregulates the expression of ginsenoside biosynthetic genes, including *FPS*, *SE*, and *UGT*, to facilitate ginsenoside production in ginseng [22,23,35,36,37,38]. This MeJA-induced culture system has also been widely used for saponin research in other medicinal plants. Moreover, MeJA-induced hairy root culture systems have been successfully established in medicinal plants, such as *Plumbago auriculata*, and transcriptome-based screening has uncovered pivotal regulatory nodes for saponin biosynthesis [39]. Quantitative expression analysis following MeJA treatment revealed that *PgWDR02* was significantly positively correlated with *SE2_4*, whereas *PgWDR21* showed a significant negative correlation with *DS_3*, *PgURT94*, and *SS_1*. In contrast, *PgWDR19* and *PgWDR29* exhibited no statistically significant correlations with the detected biosynthetic enzyme genes, with only weak correlative trends. These results imply the functional divergence of WDR paralogs during plant secondary metabolism [26,29]. Notably, none of these five candidate genes showed obvious relevance to protopanaxadiol-type ginsenoside accumulation, and we speculate that they preferentially regulate protopanaxatriol-type ginsenoside biosynthesis, which is consistent with the functional divergence of regulatory genes in different ginsenoside metabolic branches [19,20]. According to in silico subcellular localization prediction, *PgWDR24* is putatively a nucleus-localized protein. *PgWDR24* was significantly positively correlated with three rate-limiting genes (*UGT74AE2*, *FPS_22*, and *SE2_4*) but extremely negatively correlated with five protopanaxadiol-type ginsenosides (Rb1, Rb2, Rb3, Rc, and Rd), forming an atypical correlation in which elevated enzyme expression coincides with reduced end-product accumulation. We tentatively hypothesized that *PgWDR24* accelerates the transcription of upstream skeleton biosynthetic enzymes and redirects metabolic flux away from the protopanaxadiol pathway toward protopanaxatriol synthesis or other non-saponin terpenoid branches, thereby indirectly suppressing protopanaxadiol-type ginsenoside formation via metabolic flux diversion. This speculative flux-redistribution model needs further experimental testing. This diversion-based regulatory mode is analogous to the function of *SmMYC2* in *Salvia miltiorrhiza* and *PnNAC* in *Panax notoginseng*, as reported in previous studies [38,40,41]. Unlike canonical positive modulators such as *Arabidopsis TTG1* [7], rice *OsTTG1* [8], and fig *FcTTG1* [42], *PgWDR24* executes dual-directional metabolic regulation, indicating lineage-specific functional specialization of ginseng WDR proteins [40] in terpenoid metabolism and expanding the functional diversity of plant WD40-repeat proteins [20,41]. In contrast to the sharply fluctuating expression of *PgWDR19*, *PgWDR21*, *PgWDR22*, and *PgWDR24* displayed a mild expression trend of slight elevation at early MeJA-treatment time points, followed by a moderate decline, although these fluctuations did not reach statistical significance. This steady expression signature defines *PgWDR24* as a homeostatic fine-tuner that reshapes terpenoid flux via mild modulation of downstream enzyme genes to avoid excessive accumulation of protopanaxadiol-type ginsenosides and maintain intracellular metabolic equilibrium [43]. WD40 proteins are known to regulate flux partitioning in plant terpenoid and flavonoid metabolism, and this regulatory mode is consistent with the feedback balance property of the JAZ-MYC2 signaling cascade [22,23].

Our current research only clarifies the regulatory characteristics of *PgWDR24* at the transcriptional level, without in-depth functional verification via transgenic manipulation or protein interaction identification. Future studies will employ gene overexpression, yeast two-hybrid, and dual-luciferase reporter assays to identify the interacting partners and direct targets of *PgWDR24* and ultimately construct an intact regulatory cascade of MeJA-*PgWDR24*-key enzymes–protopanaxadiol ginsenosides to supply elite genetic resources for ginseng quality improvement and metabolic engineering.

## 5. Conclusions

In this study, 29 *PgWDR* genes were first identified in *ginseng*, followed by systematic analyses of their phylogeny, gene structure, chromosomal distribution, promoter cis-elements, and expression patterns. The *PgWDR* family is evolutionarily conserved, with canonical gene architectures, and exhibits obvious spatiotemporal expression specificity. Most *PgWDR* promoters contain MeJA-responsive and stress-related regulatory motifs. Six candidate *PgWDR* genes associated with ginsenoside biosynthesis were screened via correlation analysis between gene expression and ginsenoside content, as well as co-expression profiling. Subsequent MeJA induction experiments revealed that *PgWDR24*, which is predicted to localize to the nucleus, was significantly positively correlated with key ginsenoside biosynthetic enzymes but negatively correlated with the accumulation of protopanaxadiol-type ginsenosides. These correlative results suggest that *PgWDR24* may act as a negative regulator in the accumulation of protopanaxadiol-type ginsenosides. Nevertheless, this hypothesis still requires further validation via functional experiments. Collectively, this work provides novel candidate genes for uncovering the molecular mechanism of ginsenoside biosynthesis and lays a theoretical foundation for ginseng quality improvement and secondary metabolic engineering.

## Figures and Tables

**Table 1:** Protein physicochemical properties of *PgWDR* gene family in *P. ginseng*.

Gene Id	Number of Amino Acids(aa)	Molecular Weight (Da)	Theoretical pI	Instability Index	Aliphatic Index	Subcellular Location
*PgWDR01*	329	35,994.79	6.70	22.76	91.55	Peroxisome
*PgWDR02*	477	53,159.59	8.62	33.68	78.20	Nucleus
*PgWDR03*	309	34,047.39	6.82	20.12	82.27	Nucleus
*PgWDR04*	346	38,420.49	7.68	38.93	73.29	Chloroplast
*PgWDR05*	577	63,730.09	5.76	49.94	81.89	Nucleus
*PgWDR06*	569	62,690.96	6.30	42.24	81.27	Nucleus
*PgWDR07*	321	34,320.69	5.85	39.57	84.42	Cytoplasm
*PgWDR08*	645	72,058.16	5.66	49.00	84.93	Nucleus
*PgWDR09*	342	37,824.97	7.12	37.85	71.55	Cytoplasm
*PgWDR10*	790	86,322.77	7.30	40.02	84.99	Chloroplast
*PgWDR11*	1217	137,124.91	6.57	34.57	94.43	Nucleus
*PgWDR12*	616	70,130.20	5.47	24.22	67.91	Nucleus
*PgWDR13*	316	35,285.71	6.16	34.06	78.70	Cytoplasm
*PgWDR14*	300	33,162.23	6.82	35.79	74.30	Cytoplasm
*PgWDR15*	886	98,248.91	6.41	37.99	88.70	Cytoplasm
*PgWDR16*	908	102,373.75	4.85	32.05	85.36	Endoplasmic Reticulum
*PgWDR17*	377	40,964.16	6.66	36.70	81.43	Nucleus
*PgWDR18*	950	105,439.80	6.83	42.19	90.05	Chloroplast
*PgWDR19*	767	85,218.92	8.77	53.65	53.55	Nucleus
*PgWDR20*	380	41,716.89	7.13	26.46	78.50	Nucleus
*PgWDR21*	484	53,709.93	9.23	39.73	69.73	Nucleus
*PgWDR22*	390	44,426.54	6.02	38.63	81.67	Cytoplasm
*PgWDR23*	765	83,327.14	7.04	36.64	84.25	Chloroplast
*PgWDR24*	594	67,179.31	6.56	42.70	64.53	Nucleus
*PgWDR25*	527	57,660.41	5.87	30.41	83.85	Cytoplasm
*PgWDR26*	420	45,404.88	8.43	28.59	83.31	Cytoplasm
*PgWDR27*	211	23,271.26	5.98	39.23	80.90	Cytoplasm
*PgWDR28*	316	35,560.89	5.54	32.80	80.79	Chloroplast
*PgWDR29*	350	38,628.99	4.91	34.08	73.23	Nucleus

## Data Availability

The original contributions presented in this study are included in the article/Supplementary Materials. Further inquiries can be directed to the corresponding authors.

## Supplementary Materials

The following supporting information can be downloaded at: https://www.mdpi.com/article/10.3390/biology15171516/s1, Table S1. Basic information of PgWDR gene family in ginseng; Table S2. The TPM expressions of PgWDR genes in the 4 different ages (5, 12, 18, 25 years old) of ginseng roots, 14 different organs of 4-year-old ginseng, and 42 farmer cultivars of 4-year-old ginseng roots. Table S3. Primer information used for quantitative real-time PCR (qRT-PCR) in this study. Table S4. Summary of two gene sets used for candidate PgWDR gene screening.
